# Real-world outcomes of teriflunomide in relapsing–remitting multiple sclerosis: a prospective cohort study

**DOI:** 10.1007/s00415-022-11118-7

**Published:** 2022-04-11

**Authors:** Yao Zhang, Hexiang Yin, Dingding Zhang, Yan Xu, Bin Peng, Liying Cui

**Affiliations:** 1 Center of Multiple Sclerosis and Related Disorders, Peking Union Medical College Hospital, Beijing, People’s Republic of China; 2 Department of Neurology, Peking Union Medical College Hospital, Peking Union Medical College & Chinese Academy of Medical Sciences, Beijing, 100730 People’s Republic of China; 3 Medical Research Center, Peking Union Medical College Hospital, Peking Union Medical College & Chinese Academy of Medical Sciences, Beijing, People’s Republic of China; 4 Neurosciences Center, Chinese Academy of Medical Sciences, Beijing, People’s Republic of China

**Keywords:** Multiple sclerosis, Teriflunomide, Real-world, No evidence of disease activity (NEDA), Efficacy, Safety

## Abstract

**Objectives:**

To explore efficacy, risk factors, safety, and persistence of teriflunomide in relapsing–remitting multiple sclerosis (RRMS) cohort.

**Methods:**

This prospective, observational cohort study included 217 consecutive teriflunomide treated RRMS patients, 192 of which with at least 3-month persistence on teriflunomide were included in effectiveness and risk factor analyses. Multivariate Cox proportional regression analysis was performed to identify factors associated with failure of no evidence of disease activity (NEDA) 3.

**Results:**

At baseline 82% patients were treatment naïve while 18.0% interferon-β1b treated patients had stopped treatments for more than 1 year. After treatment, 79.0% patients achieved NEDA 3 at 12-month, mean annualized relapse rate (ARR) reduced significantly (0.79 ± 0.80 vs 0.16 ± 0.70; *P* < 0.001), and mean expanded disability status score (EDSS) remained stable (1.40 ± 1.67 vs 1.56 ± 1.88; *P* > 0.05)*.* Male sex (hazard ratio [HR] 1.856; 95% confidence interval [CI] 1.118–3.082, *P* < 0.05), baseline EDSS score ≥ 4 (HR 2.682; 95% CI 1.375–5.231, *P* < 0.01), and frequent relapses before treatment (HR 3.056; 95% CI 1.737–5.377, *P* < 0.01) were independent factors significantly associated with failure of NEDA 3. The most frequent adverse events (AEs) were hair thinning, alanine aminotransferase (ALT) elevation, and leukopenia, the latter two most commonly lead to teriflunomide discontinuation during the first 3 months. Persistence rates at 6, 12, and 24 months after teriflunomide initiation were 86.9%, 72.4%, and 52.8%, respectively.

**Conclusions:**

Our results support efficacy and tolerability of teriflunomide for treatment-naïve RRMS patients in real-world practice. Female patients, patients with less relapses and less disability before treatment are most likely to benefit from teriflunomide treatment.

## Introduction

Multiple sclerosis (MS), an immune-mediated chronic demyelinating disease of the central nervous system, primarily manifests as recurrent attacks during early stage disease, followed by progressive disability [1]. Recently, numerous disease-modifying therapies (DMTs) have been approved to successfully reduce relapse occurrence and slow progression to permanent disability in patients with MS. These DMTs provide better opportunities for personalized treatments, but detailed knowledge regarding how best to tailor therapy in practice remains lacking [2]. Moreover, no evidence of disease activity (NEDA), defined as an absence of relapses, disability progression lasting at least 3 months and no new MRI lesions, now become a new goal and outcome measure for MS treatment. [3]

Teriflunomide (Aubagio, Paris, Sanofi Genzyme), an once-daily, oral immunomodulatory agent, selectively and reversibly inhibits the mitochondrial enzyme dihydroorotate dehydrogenase, blocking de novo pyrimidine synthesis and reducing autoreactive T lymphocyte proliferation [4–6]. Teriflunomide efficacy and safety have been consistently demonstrated in both pivotal phase 3 randomized control trials (RCTs) [7, 8] and long-term extension studies [9, 10]. Teriflunomide has been reported to have superior efficacy compared with placebo in reducing relapses, preventing disability accumulation, and improving MRI outcomes with respect to brain lesions and atrophy measures [7, 8, 11]. The teriflunomide safety profile is manageable, with no significant changes in immune surveillance [7–9]. Most side effects are self-limiting, of mild to moderate intensity, and rarely lead to treatment discontinuation [12].

Real-world studies are important complements for RCTs, because they include an unselected patient population treated during routine clinical practice and, therefore, may provide more generalizable data. Several recently published real-world studies have confirmed the efficacy, tolerability, satisfaction, and persistence of teriflunomide treatment in patients with MS [13–16]. However, few studies have examined NEDA or predictors of treatment response, which are important factors when determining personalized treatment decisions. In addition, published post-marketing investigations indicate that teriflunomide is more commonly prescribed to male patients due to pregnancy-related safety concerns; however, evidence supporting this preferential use in male patients is less robust than general efficacy and safety data [17]. Therefore, in the present study, we collected data on the efficacy, safety, and persistence of teriflunomide treatment in a real-world patient cohort to identify demographic and clinical predictors associated with NEDA 3 failure.

## Methods

### Study population

**Fig. 1:**
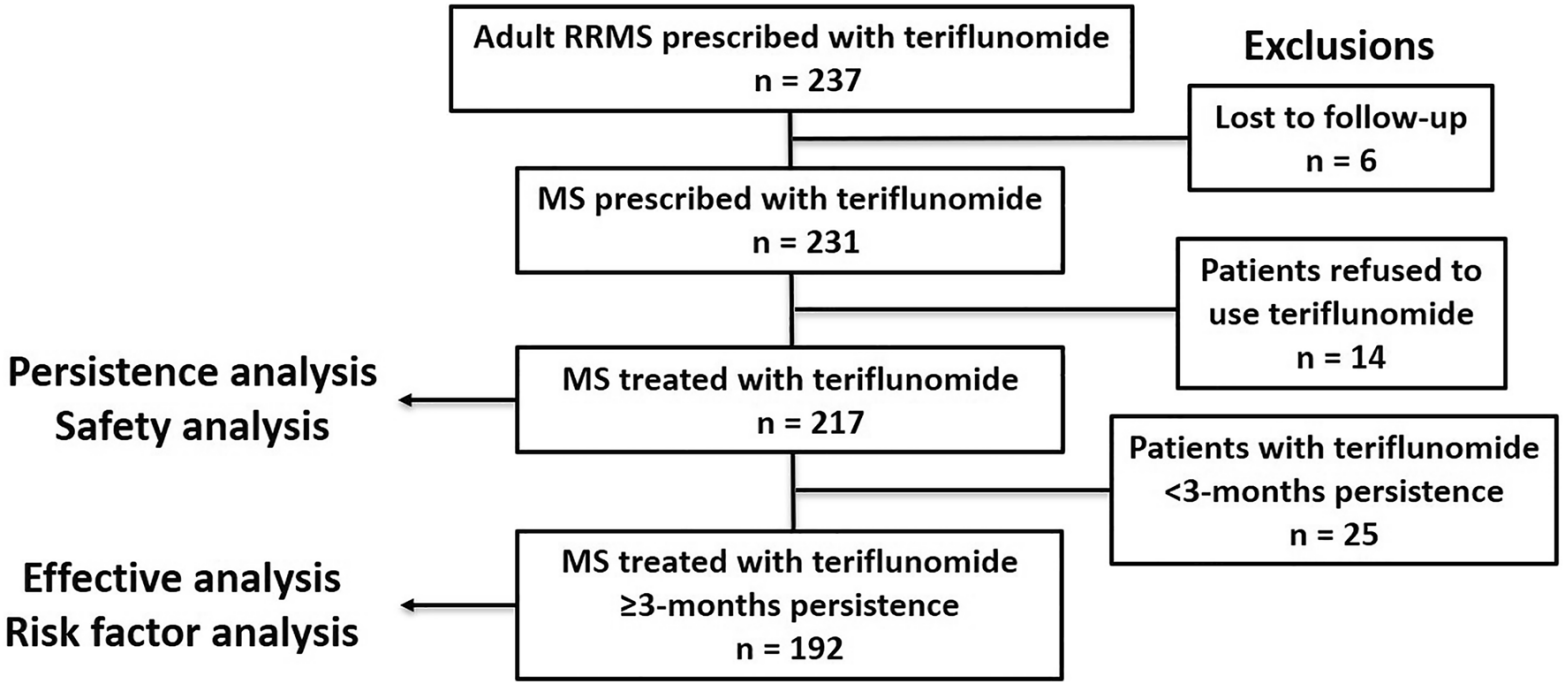
Flow chart of data processing and exclusions. *MS* Multiple sclerosis, *RRMS* relapsing remitting multiple sclerosis

This prospective, observational, cohort study used the MSNMOBase, a hospital-based electronic database established in 2011 [18, 19] to collect data on consecutive patients with MS and related disorders. Patients included in this database were followed up at least semi-annually, regardless of the clinical disease course. Before the censoring date (May 2021), 223 patients with relapsing–remitting MS (RRMS) who were treated with teriflunomide were included in the study. Teriflunomide was administered according to the approved label instructions (14 mg, once per day). All patients met the 2017 revised McDonald diagnostic criteria for MS [20]. After excluding patients lost to follow-up (*n* = 6), 217 patients were included in the baseline, persistence, and safety analyses. Only 192 patients with a minimum of 3 months of persistence on teriflunomide treatment were included in the final effectiveness and risk factor statistical analysis (Fig. 1). All patients in our study gave their informed consent prior to their inclusion in the study. This study was approved by the ethics committee of Peking Union Medical College Hospital and the approval number was ZS-1041.

### Data collection

The following data were extracted from the MSNMOBase on May 1, 2021. The following data were collected for each patient at teriflunomide initiation (defined as “baseline”): demographic data, dates of disease onset, dates of pre-treatment relapses, symptoms of previous attacks, expanded disability status scale (EDSS) scores, brain and spinal MRI outcomes, cerebrospinal fluid oligoclonal bands (OBs), previous treatment initiation dates, previous treatment termination dates, and reasons for previous treatment discontinuation.

**Fig. 2:**
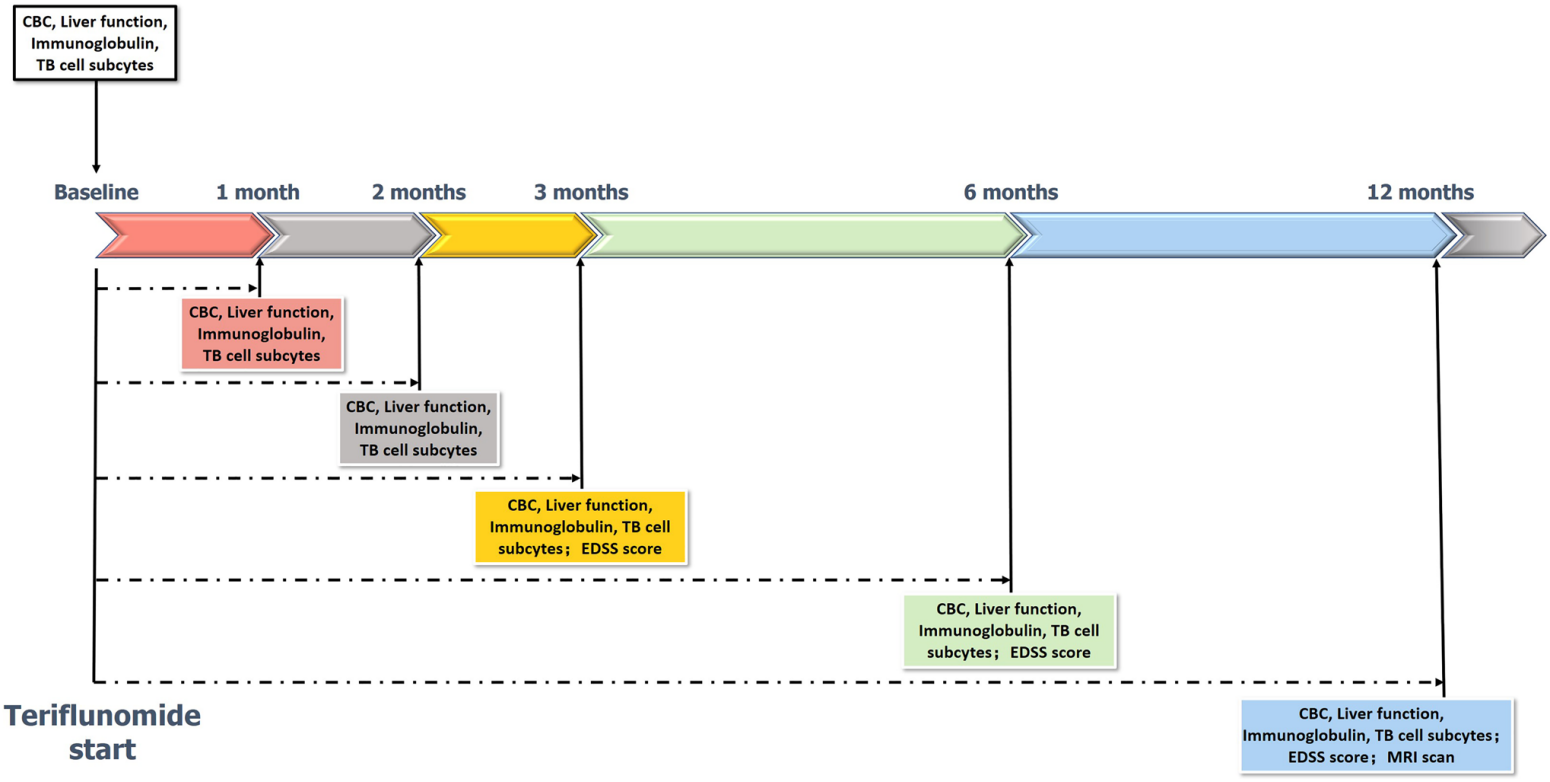
Follow-up visit schedule before and after teriflunomide start. *CBC* Complete blood counts, *EDSS* Expanded Disability Status Score, *MS* multiple sclerosis

Follow-up began 4 weeks after teriflunomide treatment initiation, and follow-up visits were scheduled every month for 3 months and then at 6-month intervals until the censoring date. Follow-up data were collected at routine clinical visits starting at baseline and continuing through the last available visit or until teriflunomide discontinuation. The dates and clinical manifestations of relapses experienced during teriflunomide treatment, EDSS scores, and adverse events (AEs) were collected at each visit, and the dates and reasons for teriflunomide discontinuation were recorded. Blood tests, including complete blood counts and liver function tests, were scheduled for each visit. MRI studies were conducted using a 3.0-T scanner with a 3-mm slice thickness (Fig. 2).

### Definitions and outcomes

Regions of the central nervous system involved at MS onset were categorized as optic nerve, cerebellum/brainstem, spinal cord, or cerebrum. Recovery from the first attack was classified as either complete (absence of neurological signs compared to baseline, EDSS = 0 or 1.0) or incomplete (partial recovery or failure to recover, persistence of neurological signs compared to baseline, EDSS ≥ 2.0). A MS relapse was defined as the appearance of any new MS-related symptoms or signs or the worsening of any existing symptoms or signs persisting for at least 24 h, in the absence of concurrent illness or fever, and occurring at least 30 days after a previous relapse [21]. Patients who experienced at least two attacks 1 year before treatment onset or at least three attacks 2 years before treatment onset were defined as having frequent relapses before treatment. Disability was assessed by Neurostatus-certified MS specialists. Confirmed disability worsening (CDW) was defined as an increase of ≥ 1.0 points in EDSS score from baseline (or ≥ 0.5 points for patients with baseline EDSS scores ≥ 5.5) that persisted over a period of ≥ 12 weeks.

The primary aim of the study was to evaluate the proportion of patients who achieved NEDA 3 over 12 months on teriflunomide treatment and identify risk factors associated with the failure to achieve NEDA 3. NEDA 3 was defined as a composite that consisted of (a) the absence of clinical relapses; (b) no CDW sustained for 12 weeks (as measured by EDSS); and (c) no new or enlarging T2 or T1 gadolinium-enhancing lesions on annual MRI. The secondary outcomes were the persistence rate and the proportion of patients with AEs, using European Medical Agency definitions of AEs.

### Statistical analysis

Descriptive variables are presented as the percentage (%), mean with standard deviation (SD), or median with interquartile range (IQR). Cox proportional regression analysis was used to investigate the following factors as potential predictors of failure to achieve NEDA 3: sex, age at disease onset, disease course at treatment start, EDSS score at treatment start, involved nervous system regions at disease onset, recovery from the first attack, frequent relapses before treatment, the number of relapses during the first 2 years after disease onset, AEs, OB, relapse before treatment, and whether teriflunomide was the first-line treatment. First, an univariate model was applied including potential predictors, and the results are expressed as the hazard ratio (HR) and 95% confidence interval (CI). All variables with *P* < 0.05 in the univariate analysis were included in the multivariate analysis. The variables were inserted one by one and removed if they were found to have lost significance. Variables with *P* < 0.05 were maintained in the analysis. Factors with *P* < 0.05 in the multivariate analysis were considered significant.

All statistical analyses were performed using SPSS software, version 26.0 (SPSS Inc., Chicago, IL, USA).

## Results

### Cohort characteristics

The baseline demographic and clinical characteristics of the 217 patients included in the baseline analysis are summarized in Table 1. Among these patients, 140 (64.5%) were women, and the median (IQR) age at disease onset was 29.7 (23.8, 37.4) years. The median (IQR) EDSS score at baseline was 1.0 (0.00, 2.00), and 200 (92.2%) patients had baseline EDSS scores less than 4.0. The most commonly isolated regions involved at disease onset were the spinal cord (68 [31.3%]) and cerebrum (62 [28.6%]). A total of 111 (51.2%) patients recovered completely from their first attack, whereas others had either partial recovery (82 [37.8%]) or failed to recover (17 [7.8%]). Before starting teriflunomide treatment, 84 (38.7%) patients experienced no relapses, whereas 31 (14.3%) patients had frequent relapses (see definitions), and 25 (11.5%) patients had at least two relapses during the first 2 years after disease onset.

**Table 1:** Demographic and clinical characteristics of patients

Characteristics	
No. of patients	217
Female, no. (%)	140 (64.5)
Age at onset, median (IQR), years	29.7 (23.8, 37.4)
Disease duration before treatment, median (IQR), years	2.74 (0.64, 6.52)
EDSS score at treatment start, median (IQR)	1.00 (0.00, 2.00)
EDSS score < 4, no. (%)	200 (92.2)
EDSS score ≥ 4, no. (%)	17 (7.8)
Regions involved at onset, no. (%)	
Isolated optic nerve	33 (15.2)
Isolated brainstem/cerebellum	43 (19.8)
Isolated spinal cord	68 (31.3)
Isolated cerebrum	62 (28.6)
Poly-system onset	12 (5.5)
Recovery from the first attack, no. (%)	
Complete recovery	111 (51.2)
Partial recovery	82 (37.8)
Failed to recover	17 (7.8)
Oligoclonal band positive, no. (%)	157 (72.4)
Pre-treatment relapses, no. (%)	
0	84 (38.7)
≥ 1	133 (61.3)
Frequent relapses before treatment^a^, no. (%)	31 (14.3)
≥ 2 relapses during the first 2 years, no. (%)	25 (11.5)
Treatment before teriflunomide initiation, no. (%)	
None	178 (82.0)
Beterferon β-1b	39 (18.0)

*EDSS* Expanded Disability Status Scale, *IQR* interquartile range

^a^Patients who had at least 2 attacks 1 year before treatment or at least 3 attacks 2 years before treatment were considered had frequent relapses before treatment

Before teriflunomide initiation, although 39 (18.0%) patients had received DMTs (interferon β1-b), all of them had discontinued their treatments for more than 1 year and the treatment duration was not long (6.87 [2.63, 21.83] months), because interferon β1-b was not available in mainland China in December 2016. The median (IQR) teriflunomide treatment follow-up time was 18.3 (9.9, 24.2) months.

### Efficacy

After excluding 25 patients treated with teriflunomide for less than 3 months, 192 patients were included in the effectiveness analysis. The mean annualized relapse rate (ARR) reduced from 0.79 ± 0.80 at 1 year before treatment to 0.16 ± 0.70 during treatment (− 79.7%, *P* < 0.001). The mean EDSS scores at treatment initiation and last follow-up were 1.40 ± 1.67 and 1.56 ± 1.88, respectively (*P* = 0.658). During the teriflunomide follow-up period, 67 (65.1%) patients achieved NEDA 3, 16 (8.3%) patients experienced clinical relapses, 30 (15.6%) patients had new or enlarging T2 or T1 gadolinium-enhancing lesions on annual MRI, and 25 (13.0%) patients experienced CDW. The proportion of patients with NEDA 3 over 12 months was 79.0%.

The univariate analysis identified male sex (HR 1.635; 95% CI 1.002–2.669, *P* = 0.049), EDSS score ≥ 4 at treatment start (HR 2.237; 95% CI 1.167–4.289, *P* = 0.015), frequent relapses before treatment (HR 2.926; 95% CI 1.669–5.131, *P* = 0.000), and ≥ 2 relapses during the first 2 years after disease onset (HR 2.691; 95% CI 1.460–4.957, *P* = 0.001) as significantly associated with the failure to achieve NEDA 3 (Table 2). The multivariate analysis confirmed that male sex (HR 1.856; 95% CI 1.118–3.082, *P* = 0.017), EDSS score ≥ 4 at treatment start (HR 2.682; 95% CI 1.375–5.231, *P* = 0.004), and frequent relapses before treatment (HR 3.056; 95% CI 1.737–5.377, *P* = 0.000) were independently and significantly associated with the failure to achieve NEDA 3 (Table 2).

**Table 2:** Univariate and multivariate analyses of predictors of NEDA 3 failure

Factors	Univariate analyses		Multivariate analyses	
	HR (95% CI)	*P* value	HR (95% CI)	*P* value
Male	1.635 (1.002, 2.669)	**0.049**	1.856 (1.118, 3.082)	**0.017**
Age at onset	0.981 (0.958, 1.005)	0.124		
Disease course at treatment start	1.032 (0.991, 1.075)	0.128		
Baseline EDSS ≥ 4	2.237 (1.167, 4.289)	**0.015**	2.682 (1.375, 5.231)	**0.004**
Regions involved at onset (yes vs no)				
Isolated optic nerve	1.632 (0.912, 2.919)	0.099		
Isolated brainstem/cerebellum	0.690 (0.341, 1.398)	0.303		
Isolated spinal cord	0.886 (0.530, 1.481)	0.644		
Isolated cerebrum	0.989 (0.576, 1.700)	0.969		
Poly-system onset	1.143 (0.358, 3.650)	0.821		
Recovery from 1st attack				
Complete recovery	0.995 (0.614, 1.610)	0.982		
Incomplete recovery	0.998 (0.610, 1.634)	0.994		
No recovery	1.023 (0.440, 2.375)	0.958		
Positive SOB	0.785 (0.395, 1.562)	0.491		
Relapse before treatment	1.548 (0.921, 2.601)	0.099		
Frequent relapses before treatment^a^	2.926 (1.669, 5.131)	**0.000**	3.056 (1.737, 5.377)	**0.000**
≥ 2 relapses during the first 2 years	2.691 (1.460, 4.957)	**0.001**		
Teriflunomide dose reduction	0.912 (0.546, 1.521)	0.723		
Teriflunomide as first treatment	1.057 (0.637, 1.756)	0.830		

Bold values indicate the statistical significance

*ALT* alanine aminotransferase, *CI* confidence interval, *EDSS* Expanded Disability Status Scale, *HR* hazard ratio, *SOB* Specific Oligoclonal Band positive

^a^Patients who had at least 2 attacks 1 year before treatment or at least 3 attacks 2 years before treatment were considered had frequent relapses before treatment

### Persistence and safety

At database lock (May 2021), 142 (65.4%) patients remained on teriflunomide treatment after a median (IQR) treatment follow-up time of 18.3 (9.9, 24.2) months, whereas 75 (34.6%) patients discontinued teriflunomide after a median (IQR) treatment duration of 8.9 (2.5, 13.8) months. Overall, at 6, 12, and 24 months after treatment onset, the percentages of patients who persisted on teriflunomide treatment were 86.9%, 72.4%, and 52.8%, respectively. The reasons cited for treatment discontinuation among the 75 patients who discontinued treatment included persistent disease activity despite treatment for 28 (37.3%) patients; poor tolerability for 18 (24.0%) patients; inability to afford teriflunomide treatment for 13 (17.3%) patients; failure to obtain medication due to the coronavirus disease 2019 (COVID-19) pandemic for 10 (13.3%) patients; participation in clinical trials for 4 (5.3%) patients, and pregnancy preparation for 2 (2.7%) patients. Early discontinuation, defined as treatment discontinued within 3 months of initiation, occurred for 21 (28.0%) patients, primarily driven by AEs (*n* = 10, 47.6%), teriflunomide cost (*n* = 6, 28.6%), and participation in clinical trials (*n* = 4, 19.0%) rather than persistent disease activity (*n* = 1, 1.3%).

In the safety analysis set, which comprised 217 patients, 151 patients (69.6%) reported a total of 246 AEs, and only 18 (8.3%) patients discontinued teriflunomide due to AEs. All AEs reported by patients are shown in Table 3. The frequencies of the following AEs were greater than 10%: hair thinning or decreased hair density (*n* = 101, 46.5%), alanine aminotransferase (ALT) elevation (*n* = 44, 20.3%), and leukopenia (*n* = 39, 18.0%). Serious AEs leading to hospital admissions occurred in three patients, including pneumonia (*n* = 2, 0.9%) and urinary tract infection (*n* = 1, 0.5%).

**Table 3:** Adverse events of teriflunomide

Adverse events	*N*	%
Type of adverse events		
Hair thinning or decreased hair density	101	46.5
Increase of liver enzymes	44	20.3
Leukopenia	39	18.0
Diarrhea	14	6.5
Skin rash or urticaria	13	6.0
Weight loss	12	5.5
Nausea and vomiting	4	1.8
Headache	4	1.8
Arterial hypertension	3	1.4
Recurrent bladder infections	3	1.4
Muscle pain	2	0.9
Pneumonia	2	0.9
Arthralgia	1	0.5
Palpitation	1	0.5
Aphthous ulcer	1	0.5
Nasosinusitis	1	0.5
CK elevation	1	0.5
Total number of patients with adverse events	151	69.6
Dose reduction of teriflunomide due to adverse events	57	26.3
Discontinuation of teriflunomide due to adverse events	18	8.3
Hospital admission due to adverse events	3	1.4

*CK* creatine kinase

## Discussion

This prospective, observational cohort study provides insights into the effectiveness, persistence, and safety of teriflunomide in RRMS and risk factors associated with treatment failure in real-world practice. Different from previous studies, this study focused on the naïve patients and we identified male sex, EDSS scores ≥ 4 and frequent relapses during the 2 years before treatment as independent risk factors for failure to achieve NEDA 3. These findings will assist neurologists in identifying promising candidates for teriflunomide and making appropriate personalized treatment decisions.

In this study, all the patients admitted were included unselectively and were consistently treated with teriflunomide. Different from previous real-world studies, all the patients included in this study were not on any DMT at least for 1 year prior to teriflunomide initiation. Since the efficacy of teriflunomide was reported in subgroup analyses of pivotal studies to be different between patients with and without prior DMT [22], the data of our study might better represent the effectiveness and safety data of teriflunomide in treatment-naïve patients.

Based on the results of a phase II study which found that teriflunomide began to reduce the cumulative active lesions significantly 12 weeks after its initiation [23], only patients with a minimum 3-month persistence on teriflunomide were included in the effectiveness analysis. There was no significant difference in baseline demographic and clinical characteristics between the patients excluded and included. Like previous pivotal RCTs and real-world studies [7, 8, 16], our study also found that teriflunomide significantly reduced ARR (from 0.79 at 1 year before treatment to 0.16 during treatment) and maintained stable EDSS scores.

NEDA, suggesting complete disease remission, has gained increasing attention as an important treatment goal in both clinical trials and practice recently [3, 24, 25]. In our study, the proportion of patients achieving NEDA 3 at 12 months after treatment was 79.0%. This proportion was not only higher than that of patients with placebo (11.6%) in TEMSO study [26] but also higher than that of patients with teriflunomide (46%) in Teri-RADAR study [27], further confirming the effectiveness of teriflunomide in the real world. Different from Teri-RADAR study, our study included treatment-naïve patients with shorter disease duration (2.74 vs 13.8 years) and lower baseline EDSS score (1.0 vs 3.0), which might account for the higher proportion of patients achieving NEDA 3 in our study.

In analysis of predictors associated with the failure of achieving NEDA 3, we found that male sex was an independent risk factor. Although post-marketing studies had showed propensity to prescribe teriflunomide to men rather than women, driven by safety concerns about pregnancy [28], both our study and a nationwide Danish investigation [29] including more than 3200 teriflunomide-treated patients suggested a need for more aggressive treatment to provide sufficient disease control for male patients. In addition, patients with frequent relapses (≥ 2 attacks 1 year or ≥ 3 attacks 2 years) before treatment or with EDSS score ≥ 4 at treatment initiation were less likely to achieve NEDA 3 under teriflunomide treatment in our study. This result is consistent with that from TER-Italy study, another real-world investigation including 1507 teriflunomide-treated patients and finding that patients with more pre-treatment relapses or EDSS score > 4 were more likely to experience treatment failure [30]. Therefore, both our study and the real-world investigations from Caucasian cohorts suggested a propensity for prescribing teriflunomide to female patients with less baseline disability and fewer attacks prior to treatment. These predictors of NEDA 3 failure for teriflunomide treatment would be useful for personal treatment decision-making among MS patients.

The persistence rates of teriflunomide in our cohort were 72.4% at 12 months and 52.8% at 24 months, which is comparable to the result of previous real-world investigations from Denmark (72.7%, 12 months) [29] and France (60%, 24 months) [31]. Like these western studies, the most common reason for teriflunomide discontinuation in our cohort was insufficient effectiveness (37.3%), suggesting teriflunomide is a mild DMT for disease control in both Asian and western populations.

The types of AEs reported by our patients were consistent with that of pivotal RCTs [7, 8] and other real-world studies [16, 28, 29], whereas the incidence different. Similar to previous real-world investigations [13, 29], 69.6% of our patients experienced AEs, whereas only 8.3% of patients discontinued treatment due to AEs. In addition, unlike other western studies, the proportions of patients with elevated ALT and leukopenia were particularly high (> 10%) in our study. Ethnic heterogeneity and differences in teriflunomide metabolism due to *ABCG2* polymorphisms in Asian populations might explain this difference [32]. Early teriflunomide discontinuation (< 3-month treatment) in our study was primarily driven by AEs, most commonly ALT elevation and leukopenia. Therefore, the regular monitoring of liver function and blood cell counts remains necessary during the teriflunomide treatment, especially during the first 3 months after treatment initiation.

This study has some limitations. First, this is a single-arm, open-label study, which may produce selection bias. However, this bias should be low, because teriflunomide was the only available DMT for MS in China during July 2018 to January 2020 and physicians did not have other options. Second, due to the low prevalence of MS in China, the sample size of this cohort study was smaller than western studies. However, the primary predictors associated with failure of teriflunomide treatment identified in this study were similar to those identified by other large-scale, real-world investigations [30, 31]. However, an additional larger, multicenter, prospective cohort study remains necessary to further explore the predictors of response to teriflunomide.

Despite these limitations, this real-world study supports that teriflunomide is an effective and generally well-tolerated DMT for RRMS. Importantly, we provided guidance regarding which treatment-naïve patients are likely to benefit from teriflunomide: women with mild disease activity (≤ 1 attack in 1 year or ≤ 2 attacks in 2 years before treatment) and EDSS score < 4 at treatment initiation.

## Data Availability

The data that support the results of this study are available from the corresponding author upon reasonable request.

## Abbreviations

AEs: Adverse events
ALT: Alanine aminotransferase
ARR: Annualized relapse rate
MS: Multiple sclerosis
NEDA: No evidence of disease activity
RRMS: Relapsing–remitting multiple sclerosis
EDSS: Expanded disability status scale
MRI: Magnetic resonance imaging
SD: Standard deviation
IQR: Interquartile range
HR: Hazard ratio
CI: Confidence intervals
